# Retraction notice for: “Polydatin protects H9c2 cells from hypoxia-induced injury via up-regulating long non-coding RNA DGCR5” [Braz J Med Biol Res (2019) 52(12): e8834]

**DOI:** 10.1590/1414-431X20208834retraction

**Published:** 2020-08-03

Jinhua Daihttps://orcid.org/0000-0001-9371-5301^1^, Jianbo Mahttps://orcid.org/0000-0003-0046-0835^1^, Yufeng Liaohttps://orcid.org/0000-0003-4086-7744^1^, Xianhai Luohttps://orcid.org/0000-0002-7336-4468^2^, and Guofang Chenhttps://orcid.org/0000-0002-6951-5980^3^

^1^Department of Clinical Laboratory, Hwa Mei Hospital, University of Chinese Academy of Sciences (Ningbo No. 2 Hospital), Ningbo, Zhejiang, China

^2^Department of Clinical Laboratory, Ningbo Kangning Hospital, Ningbo Mental Health Center, Ningbo, Zhejiang, China

^3^Department of Cardiology, Hwa Mei Hospital, University of Chinese Academy of Sciences (Ningbo No. 2 Hospital), Ningbo, Zhejiang, China

Correspondence: Xianhai Luo: <xianhai01@sina.com> | Guofang Chen: <chen09gf@sina.com>

**Retraction for:** Braz J Med Biol Res | doi: http://10.1590/1414-431X20198834 | PMID: 31826181 | PMCID: PMC6903803

The Brazilian Journal of Medical and Biological Research received a request from the authors to withdraw this manuscript. Meanwhile, the Editors became aware of a denouncement published by independent journalists from the “For Better Science” website including this paper. This denouncement consisted of potential data falsification and/or inaccuracy of results in western blots and flow cytometry plots.

As per consensus between the Editors-in-Chief of the Brazilian Journal of Medical and Biological Research (BJMBR) and the Authors, the article titled “Polydatin protects H9c2 cells from hypoxia-induced injury via up-regulating long non-coding RNA DGCR5” that was published in year 2019, volume 52, issue 12, (Epub Dec 5, 2019) has been retracted.

